# Chronic non-communicable disease burden among reproductive-age women in India: evidence from recent demographic and health survey

**DOI:** 10.1186/s12905-023-02171-z

**Published:** 2023-01-17

**Authors:** Shri Kant Singh, Kirti Chauhan, Parul Puri

**Affiliations:** Department of Survey Research and Data Analytics, International Institute for Population Sciences, Mumbai, Maharashtra India

**Keywords:** Chronic diseases, India, Multimorbidity, Non-communicable diseases, Reproductive-aged women

## Abstract

**Background:**

Chronic disease burden among women leads to various detrimental consequences, impacting women’s health throughout their life course and off-springs. The present study explores the chronic disease profile among reproductive-aged women and analyzes the effects of various covariates on multimorbidity among reproductive-aged women in India. Here, multimorbidity is defined as an individual suffering from two or more chronic conditions.

**Methods:**

The present study employed the most recent National Family Health Survey round, 2019–2021. The study utilized information on 695,707 non-pregnant women aged 15–49 years. The study used descriptive, bivariate, and multivariable ordered logistic regression analysis to explore the burden of chronic non-communicable diseases and multimorbidity.

**Results:**

The mean age of women with single chronic condition-related morbidity is 30 years, whereas it was 35 years for those with multimorbidity. Approximately 28% of urban women suffered from multimorbidity. Further, significant factors that affect multimorbidity include age, educational attainment, working status, marital status, parity, menopause, religion, region, wealth index, tobacco use, alcohol consumption, and dietary patterns.

**Conclusions:**

The present study hints that women in the reproductive age group are at very high risk of developing multimorbidity in India. Most of the programs and policies are focused on the elderly population in terms of awareness and facilitating them with better health services. However, right now, one should also prioritize the emerging chronic condition related to chronic conditions other than hypertension, diabetes, and cancer among the study population, which is escalating as soon as women reach 30 years of age.

## Background

A rapid epidemiological transition with a change in disease burden to non-communicable diseases (NCDs) may be noticed, especially in India, where the burden of NCDs is increasing at an alarming rate [1]. In India, one in every four people has a risk of dying from an NCD (i.e., cardiovascular, stroke, cancer, diabetes, etc.) before reaching the age of 70 years [2]. Disease epidemiology suggests that NCDs transmit through the pathways of behaviour, shared patho-physiological and environmental risk factors [3]. This associative nature of NCDs has resulted in increased cases of simultaneous disease occurrence, also known as, Multimorbidity [4].

Multimorbidity (or coexisting NCDs) are more common in low-and middle-income nations, according to the World Health Organization (WHO) [5]. While multimorbidity is more common in elderly individuals, research has indicated that people under 65 years have a significant multimorbidity burden [6, 7]. According to a recent report from the Longitudinal Ageing Study in India (LASI), multimorbidity affects roughly 18% of those aged 45 years and up [8]. Categorically, women are burdened more often than men, be it diabetes, hypertension, obesity, anemia, cancer, or thyroid [9–15].

Women, in general, outlive men as they have greater life expectancies [16]. However, reporting some rise in life expectancy per decade does not necessarily indicate improved health scenario among women [4]. About 30% of the women had at least one chronic morbidity in India, and 9% had two or more morbidities [17]. When paired with reproductive and biological changes during childbearing ages, multimorbidity can lead to a declined quality of life and well-being [1, 6]. As a result, more years of life are spent in comorbid conditions. This has resulted and proven feminization of chronic diseases and multimorbidity.

Women's health in India is determined not just by biological disparities in health behaviour but also by prevailing socio-economic, cultural, and political conditions [18]. Hence, reproductive-aged women are at a higher risk of developing chronic disease and face deleterious implications including adverse pregnancy outcome [19]. Further, this impacts the life course of mothers [20–22]. Similarly, being born to a mother with any chronic disease increases the risk of undernutrition, delayed physical and cognitive progression, and perhaps developing NCDs among children [22].

The synergies of various chronic diseases can be ascertained by an individual’s age, gender, educational level, marital and working status, and behavioural factors like smoking, tobacco and alcohol consumption [3, 4, 9]. Explanatory factors at the population level include people from lower socio-economic categories, ethnicity, and reproductive characteristics such as parity and menopause [1, 4, 23]. Gender can be a significant confounding factor, as the prevalence of multimorbidity is higher in women than in men[6, 24, 25]. Women over 30 years old, with low education, who belong to affluent groups, married earlier, and women who used tobacco have a higher probability of having two or more morbidities [17]. Socio-economic status (SES) significantly affects the occurrence of multimorbidity along with lifestyle and life events among women [4, 17]. Further, women’s age was found to be associated positively with chronic diseases multimorbidity [23]. Hypertension and overweight combination have the most prevalent among women [17].

In India, various programs and policies are in place that predominantly focus on women’s health, particularly during pregnancy [1]. However, very few policies and programs focus on chronic disease management among women during their reproductive span, which does not suffice in suppressing the condition among reproductive-aged women. Programs and policies extended beyond pregnancies and birth outcomes in women’s health care are need of the hour.

NCDs are a severe public health concern in the twenty-first century because of the human suffering they cause and harm to a country’s socio-economic development. NCDs financially burden individuals and families due to the confluence of medical costs, transportation costs to and from health care facilities, time spent providing informal care, and lost productivity [22]. In addition, women are more likely to report multimorbidity than men [6, 25, 26]. Multimorbidity and disease induced mortality affect household’s functioning in terms of out-of-pocket expenditure [26–28]. Health care expenditure for low-income families due to chronic conditions has a catastrophic impact on many households and leads to increased distress in health financing [28]. Notably, in rural areas, due to increased female-headed households where men migrate for employment, mortality due to NCDs among women pushes families deeper into poverty and further lowers the socio-economic status [22]. Therefore, hampering the future generation's socioeconomic and health status.

Rather than looking at selected NCDs individually, the current study intends to find predictors of NCDs related multimorbidity in reproductive-aged women. Existing literature has examined the predictors of multimorbidity among adult and elderly populations, but very few have focused on women in the reproductive age group in India. Therefore, the present study will explore the disease profile across various population sub-groups using a recently published nationally representative data. In addition, the study would primarily look at the effect of background characteristics, including individual, household, socioeconomic and demographic factors, along with reproductive stages, including parity and menopause, on multimorbidity among reproductive-aged women in India.

## Methods

### Data

The present study utilized data collected under the fifth round of the National Family Health Survey (NFHS-5), 2019–2021, available in the public domain for legitimate research purposes. It can be obtained through https://dhsprogram.com/data/available-datasets.cfm. The survey covered a range of health-related issues, including non-communication diseases.

NFHS-5 is the second nationwide community-based survey after NFHS-4 in India to provide estimates of blood glucose levels and blood pressure in the general population. Specifically, among women aged 15–49 years and men aged 15–54 years for all the Indian States and Union Territories (UTs), and districts. Survey data consists of 724,115 women samples. After dropping 28,408 pregnant women observations, the final analysis used information on the remaining 695,707 women samples. Women generally modify their dietary and lifestyle behaviours during pregnancy; therefore, including pregnant women in the study might affect the study estimates. For the same reason, they were dropped off from the final analysis.

### Ethics statement

The present study utilizes a secondary data set from the recent NFHS-5 survey with no identifiable information on the survey participants. This dataset is available in the public domain for legitimate research purposes. Hence, there is no requirement for any additional ethical approval. The study utilizes data from a national survey conducted under the stewardship of the Ministry of Health & Family Welfare, Government of India, with the help of the International Institute for Population Sciences, Mumbai. The survey received ethical clearance from the Institutions Review Board (IRB) of the International Institute for Population Sciences, India. Additionally, the NFHS survey has taken consent from all the eligible participants age 18 & above. However, participants in the age 15–17 years required consent was taken from their parents.

### Outcome variables

The outcome of interest was the chronic disease score (CDS), computed using the information on eight non-communicable diseases available in NFHS-5. Out of these eight, four were self-reported; these included asthma, cancer, chronic heart disease, and thyroid disorders. Whereas diabetes, hypertension, obesity, and anemia were measured as amalgamating self-reported and measured diagnosis of chronic conditions. A woman was categorized as diabetic if their random blood glucose level ≥ 140 mg/dl. Women with average systolic blood pressure > 140 mmHg or average diastolic blood pressure > 89 mmHg were considered hypertensive. Obesity was measured using Quetelet Index, also known as Body Mass Index (BMI), calculated as:

$${\varvec{Body\,Mass\,Index}}=\frac{Weight\,(in\,Kgs)}{{Height}^{2} (in\,{m}^{2})}$$

A woman was considered obese if her BMI ≥ 25 (kg/m^2^) [29].

All the eight diseases were coded into binary categories of absent—‘0’ and present—‘1’. Finally, the outcome variable, i.e., chronic disease score (CDS), was generated and was further classified into three, no morbidity (women with zero chronic disease), single morbidity (women with exactly one chronic illness), and multimorbidity (women who are suffering from two or more chronic conditions simultaneously).

### Explanatory variables

The present study included three sets of explanatory variables: (1) socio-demographic and economic factors, including age (categorised into 5 years age group between 15 to 49), place of residence (categorised as urban and rural), religion (categorised as “Hindu”, “Muslim”, and “Other”), marital status (categorised as “Ever married” and “Never married”), parity (categorised as “no children”, “one child”, and “two or more”), and menopause (categorised as “yes’ and “no), working status (categorised as “yes” and “no”), and wealth index (categorised into “poorest”, “poor”, middle”, “rich”, and “richest”) (2) health behaviours; including tobacco use (categorised as “yes’ and “no”), alcohol consumption (categorised as “yes’ and “no”), dietary habits (categorised as “normal/healthy’ and “unhealthy), and (3) anthropometric indicator: waist-hip ratio (WHR) (categorised as “high risk WHR’ and “low risk WHR”).

#### Dietary Index

In NFHS, nine questions pertaining to dietary practices were asked. The frequency (frequently, occasionally, and never) of consuming nine food items in a week, namely milk/curd, pulses/beans, dark green leafy vegetables, fruits, eggs, fish, chicken/meat, fried food, and, aerated drinks were available. However, the use of MCA facilitated in making an index that combines good and bad eating habits after re-coding nine items in a unidirectional manner, such that each item measures the same concept, where “0” corresponds to those who “frequently consume, say, cereal,” which can be considered as a good eating habit. Whereas, in the case of junk/sweet/fried foods, “0” were those who “never consume junk food,” which is again a good thing. Similarly, re-coding was done for the remaining items. Prior to index computation, Cronbach's alpha ‘α’ was used to verify internal consistency between the nine features. Cronbach’s alpha measures internal consistency, that is, how closely related a set of items are as a group. Finally, using MCA, an index was generated and divided into two categories after sorting in ascending order [30]. Thus, the “index value” in the second half would be higher values which were considered as an unhealthy diet index coded as “1” and the other half as “0”. Here unhealthy diet practitioners were identified as those who consume milk/curd, pulses/beans, dark green leafy vegetables, fruits, eggs, fish, chicken/meat occasionally/never and consume fried food, and, aerated drinks occasionally/daily.

Waist-Hip Ratio (WHR), was measured using:

$$WHR=\frac{Waist\,Circumferenc\,(in\,cm)}{Hip\,Circumference\,(in\,cm)}$$

A woman is considered at a high risk of developing long-term health conditions if her WHR ≥ 0.85.

### Statistical analysis

Firstly, descriptive statistics were conducted to study the sample distribution. Further, women’s disease profile was explored using prevalence measured using:

$$\begin{aligned}&\mathbf{Prevalence}\,(\mathbf{per}1000\,\mathbf{women})\\ & =\frac{\mathrm{All\,new\,and\,existing\,cases\,during\,a\,given\,time\,period}}{\mathrm{Surveyed\,women\,during\,the\,same\,time\,period}}\,*\,1000\end{aligned}$$

In addition, bivariate analysis was used to understand the chronic disease burden among reproductive-aged women by socio-economic and demographic variables across India in 2019–2021.

In epidemiological and biomedical studies, the proportional odds model (POM) has often been used [31]. The proportionality assumption was checked using the brant’s test before further analysis. However, if the proportionality assumption does not hold, the partial proportional odds model may have been a better choice (which was not the present case) [32]. If the log odds ratio across the cut points is identical, i.e., the proportional odds assumption is satisfied, the proportional odds model is used.

Observations on the chronic condition related to multimorbidity (Y) for each woman are classified into three categories. Likewise, covariates (x_i_) denote the p-dimensional vector of covariates (i = 1, 2, …, p), containing the observation on the complete set of p explanatory variables. Accordingly, the dependency of Y on x_i_ can be expressed as:

$$\mathrm{Pr}(Y\ge {y}_{j}|{x}_{i})=1/(1+exp\left(-{\alpha }_{j}-{x}_{i}^{^{\prime}}\beta \right), j=\mathrm{0,1},2$$

Or

$$\mathrm{log}\left[\frac{Pr(Y\ge y\_j |x)}{1-Pr(Y\ge y\_j |x)}\right]=-{\alpha }_{j}-{x}_{i}^{^{\prime}}\beta , j=\mathrm{0,1},2$$

where $\mathrm{Pr}(Y\ge {y}_{j})$ is the cumulative probability of the event $Y\ge {y}_{j}$; ${\alpha }_{j}$ are the respective intercept parameters; β is a (p by 1) vector of regression coefficients corresponding to ${x}_{i}$ covariates. Results are then presented as an odds ratio (OR) with a 95% confidence interval (CI).

Statistical analysis and data visualization was performed with STATA version 15.0 (StataCorp™, Texas) and MS Excel. A *p*-value < 0.05 was considered as statistically significant for all calculations. All estimates were reported by applying appropriate sampling weights. As the data used in the study was taken from Women’s file, national women’s weights were employed in the analysis. Additional information on survey weight can be seen from national report of NFHS (Add reference of the report here).

## Results

### Description of the study population

Table 1 depicts the sample distribution of women aged 15–49 years by various background characteristics in India NFHS-5 (2019–2021).

**Table 1:** Sample distribution by background characteristics, National Family Health Survey (NFHS-5), India, 2019–2020

Continuous correlates	Mean (Standard Deviation)	(Min., Max.)
Age (in years)	30.61 (9.973)	(15, 49)
Waist-Hip Ratio	0.86 (0.150)	(0.03, 33.32)

Categorical correlates	Sample distribution (N)	Weighted percentage
*Place of residence*		
Urban	173,942	32.76
Rural	521,765	67.24
*Religion*		
Hindu	525,869	81.48
Muslim	86,140	13.33
Others	83,698	5.19
*Social group*		
Others	129,239	26.02
Other Backward Castes	134,311	42.87
Scheduled Tribe	266,321	9.26
Scheduled Castes	165,836	21.85
*Working*		
No	76,963	74.31
Yes	27,729	25.69
*Level of education*		
No education	162,651	22.67
Primary	81,896	11.78
Secondary and above	451,160	65.54
*Wealth Index*		
Poorest	142,912	18.33
Poor	153,749	19.97
Middle	145,760	20.56
Rich	134,521	20.85
Richest	118,765	20.30
*Marital status*		
Never married	181,211	24.68
Ever married	514,496	75.32
*Children ever born*		
No child	218,471	30.36
One child	90,256	13.52
Two or more children	386,980	56.11
*Menopause*		
No	66,781	96.08
Yes	27,856	3.92
*Tobacco consumption*		
No	651,086	95.90
Yes	44,621	4.10
*Alcohol consumption*		
No	682,532	99.24
Yes	13,175	0.76
*Diet*		
Healthy	345,278	49.37
Unhealthy	350,429	50.63
Total	695,707	100.00

From the results, the mean age of the sampled women is around 30 years (Min = 15; Max = 49). The mean waist-hip ratio of the sampled women was 0.86 (Min = 0.003; Max = 33.32). Further, 67% of the reproductive-aged women lived in rural India. Roughly 81% of the women in the sample were Hindu, followed by 13% of Muslim women, and the remaining belonged to other religious groups. The maximum proportion belongs to the 'other backward class (OBC) (i.e., 43%), followed by others (26%), Scheduled Castes (22%), and Scheduled Tribes (9%). There is approximately 10% of the women working. Sixty-six percent of the reproductive-aged women had a secondary or above level of education, and 12% have only primary education.

In contrast, approximately 23% of the sample population had no education. All women are approximately equally distributed across the wealth quintile. Almost 75% of the women were ever married, whereas 25% of women were never married. There is 56% of women who have two or more children ever born, followed by 30% with no child and approximately 14% of women having only one child. Roughly 4% of the women have got menopause. Approximately 4% of women consume tobacco and about a percent of women drink alcohol. Fifty-one percent of the sample followed unhealthy dietary patterns.

### Disease profile for reproductive-aged women

**Fig. 1:**
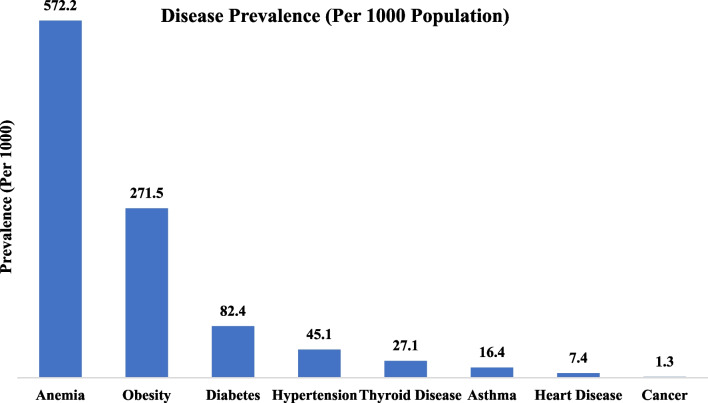
Disease Prevalence among reproductive-aged women in India, National Family Health Survey (NFHS-5), 2019–2021

Figure 1 depicts the prevalence of various chronic diseases per 1000 women in the reproductive age group. Out of the eight selected chronic conditions, anemia, obesity, diabetes, and hypertension have the highest prevalence in the selected reproductive-age women in India. Cancer, heart diseases, asthma, and thyroid diseases are the bottom four among eight NCDs chosen in terms of prevalence per 1000 women in the reproductive age group.

**Fig. 2:**
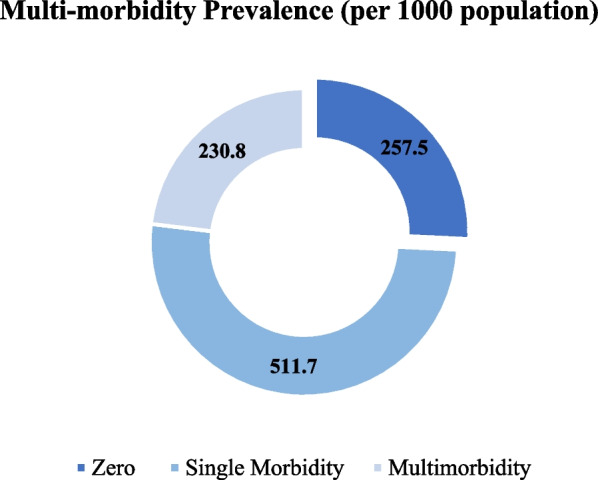
Distribution of chronic diseases among reproductive-aged women in India, National Family Health Survey (NFHS-5), 2019–2021

Figure 2 shows the distribution of chronic disease scores among reproductive-aged women. Roughly 512 per 1000 women suffer from a single chronic condition, and 258 per 1000 women suffer from two or more chronic conditions.

### Variation in the number of chronic diseases by age-group

**Fig. 3:**
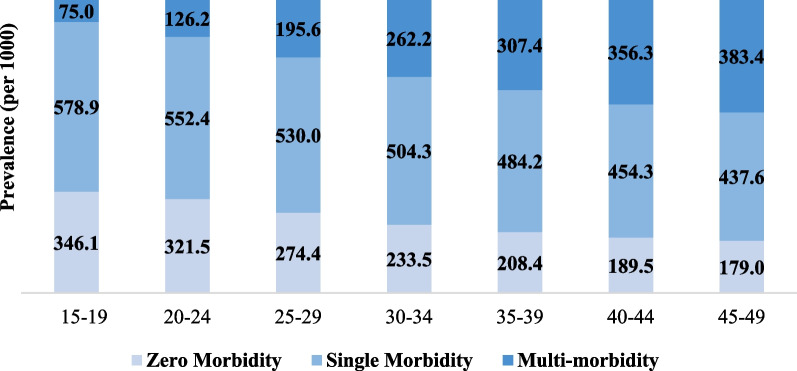
Five years age-group wise chronic disease score prevalence among reproductive-aged women in India, National Family Health Survey (NFHS-5), 2019–2021

Figure 3 shows the distribution of chronic condition-related multimorbidity across the 5 years age group. As the age group increases, the prevalence of chronic conditions related to multimorbidity rises significantly from 75 per 1000 women aged 15 to 19 years to 383 per 1000 women aged 45 to 49 years. Although in earlier ages prevalence of single morbidity is significantly higher with approximately 579 per 100 women. Besides, the prevalence of single chronic conditions decreases as the age group increases. This decrease in prevalence can be seen as the shift from single to more than two chronic condition-related morbidities among women.

### Variation in the number of chronic diseases by region

**Fig. 4:**
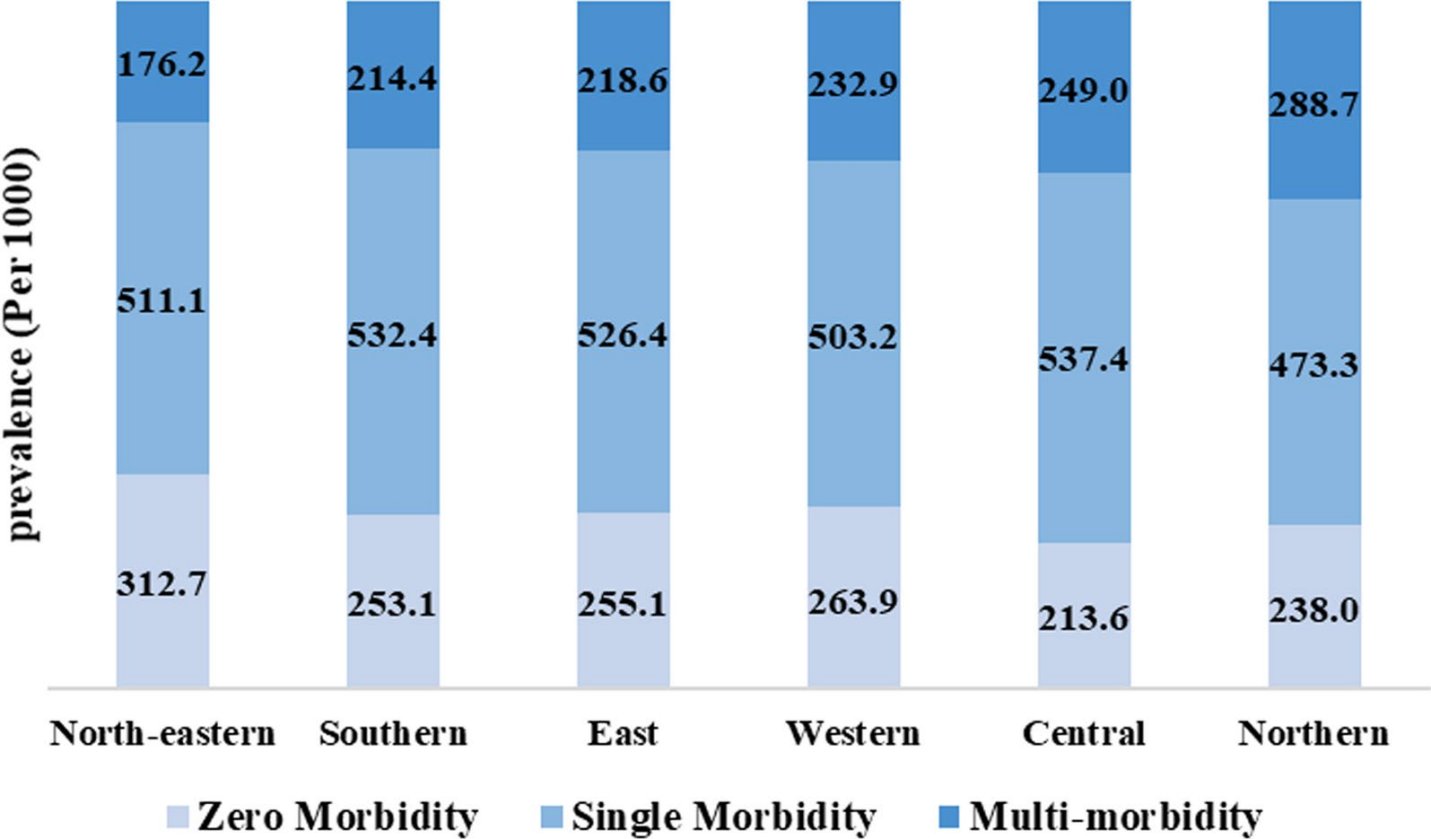
Region-wise chronic disease score prevalence among reproductive-aged women in India, National Family Health Survey (NFHS-5), 2019–2021

Figure 4 represents the region-wise multimorbidity prevalence among reproductive-aged women in India. Northern India shows the highest prevalence of chronic conditions related to multimorbidity, with 289 per 1000 women in the reproductive age group. At the same time, the lowest prevalence can be seen among those living in north-eastern India, with 176 per 1000 women of the reproductive ages. Similarly, the central region has the highest prevalence, with 537 per 1000 women, and the lowest in northern India, with 473 per 1000 women.

Table 2 shows the bivariate analysis of chronic disease scores among reproductive-aged women. The mean age of women with zero morbidity is roughly around 29 years; exactly one morbidity is 30 years, whereas it was 35 years for those with multimorbidity. The mean waist-hip ratio (WHR) for zero and single morbidity is 0.85 (SD = 0.11), whereas, for those with multimorbidity, it is around 0.89 (SD = 0.26). Approximately 48% of the urban women have one morbidity, and 28% suffer from multimorbidity. When looking at the rural women, roughly 53 and 20% are suffering from one morbidity and multimorbidity, respectively. Irrespective of the religion, nearly half of the women have one morbidity. However, the maximum proportion of women suffering from chronic disease-related multimorbidity is among those belonging to the other religion (28%), followed by Muslims (25%) and Hindus (23%).

**Table 2:** Bivariate analysis of chronic disease score among reproductive-aged women, National Family Health Survey (NFHS-5), India, 2019–2021

Continuous correlates	Chronic disease score (CDS)		
	Zero morbidity	Single morbidity	Multimorbidity
Mean age (in years)	28.53 (9.72)	29.77 (9.89)	35.34 (8.96)
Mean waist-hip ratio	0.85 (0.11)	0.85 (0.10)	0.89 (0.26)

Categorical correlates	Proportions		
*Place of residence*			
Urban	23.26	48.26	28.48
Rural	26.96	52.59	23.62
*Religion*			
Hindu	26.01	51.50	22.49
Muslim	25.27	49.85	24.88
Others	22.78	49.42	27.79
*Social group*			
Others	23.40	48.64	27.96
Other Backward Castes	27.26	50.52	22.22
Scheduled Tribe	25.60	59.20	15.2
Scheduled Castes	25.63	52.07	22.30
*Working*			
No	26.80	50.92	22.28
Yes	24.55	51.84	23.62
*Level of education*			
No education	24.43	52.19	23.38
Primary	22.81	50.37	26.82
Secondary and above	26.73	50.96	22.31
*Wealth Index*			
Poorest	27.43	57.35	15.21
Poor	27.89	53.38	18.73
Middle	26.19	51.01	22.81
Rich	24.76	48.15	27.09
Richest	22.69	46.69	30.62
*Marital status*			
Never married	34.63	55.92	9.45
Ever married	22.84	49.62	27.55
*Children ever born*			
No Child	33.33	55.30	11.37
One Child	24.20	50.79	25.01
Two or more Children	22.02	49.03	28.96
*Menopause*			
No	26.01	51.42	22.57
Yes	19.20	45.15	35.65
*Tobacco consumption*			
No	25.91	51.06	23.03
Yes	22.02	53.74	24.24
*Alcohol consumption*			
No	25.77	51.14	23.10
Yes	22.95	55.97	21.08
*Diet*			
Healthy	26.74	51.11	22.15
Unhealthy	24.73	51.24	24.04
Total	25.75	51.17	23.08

Similarly, regardless of the social category, roughly half of the women are affected with one morbidity except for those belonging to Scheduled Tribes (STs), where 59% of the women have one morbidity. The maximum proportion (i.e., 28%) of the women from the other castes suffer from multimorbidity, whereas the lowest is among STs. Irrespective of the women's working status, roughly half of the reproductive-aged women have one morbidity, and one-fifth suffer from multimorbidity. Women with no education have the maximum proportion (52%) suffering from one morbidity. However, those with primary education have the highest proportion (27%) suffering from multimorbidity, followed by no education (23%) and the lowest amongst those with at least secondary and above the level of education (22%). As the wealth index moves from richest to poorest, the proportion of women suffering from one morbidity increases significantly, i.e., from 47% among those belonging to the wealthiest quintile to 57% among those belonging to the poorest quintile. However, women from the most affluent quintile (31%) have the highest multimorbidity. And lowest being among those belonging to the poorest wealth quintile. Roughly half of the married women have one morbidity, and amongst unmarried, the proportion is a bit high at 56%. Nearly one-third proportion of married women suffers from multimorbidity. However, among unmarried women, approximately one-tenth are suffering from multimorbidity. As parity increases, the proportion of women suffering from multimorbidity increases significantly, i.e., from 11% among women with no children to 29% among women with two or more children. At the same time, women with no children have the highest proportion (55%) of suffering from one morbidity.

Furthermore, among women who have reached menopause, 35% of them were affected with multimorbidity, whereas 45% were affected with one morbidity. Tobacco-user has 53, and 24% suffer from one morbidity and multimorbidity, respectively. Similarly, among those women who consume alcohol, 56 and 21% suffered from one morbidity and multimorbidity, respectively. Lastly, among women following or consuming an unhealthy diet, 24% of them suffered from multimorbidity. However, irrespective of the diet type (i.e., healthy or unhealthy diet), half of the women have suffered from one morbidity out of the eight selected morbidities.

### Correlates of multimorbidity among reproductive-aged women in India

Table 3 shows the unadjusted ordered logistic regression analysis of chronic disease scores among reproductive-aged women regressed upon various individual and household characteristics from National Family Health Survey-5, India, 2019–2021.

**Table 3:** Unadjusted ordered logistic regression analysis of chronic disease score among reproductive-aged women, National Family Health Survey (NFHS-5), India, 2019–2021

Correlates	Unadjusted relative risk ratio (URRR)	*p*-value	95% Confidence interval
*Age (in years)*	1.05 (0.000)	< 0.001	(1.048–1.048)
*Place of residence*			
Urban (Ref.)			
Rural	0.73 (0.001)	< 0.001	(0.725–0.727)
*Religion*			
Hindu (Ref.)			
Muslim	1.09 (0.001)	< 0.001	(1.087–1.090)
Others	1.26 (0.001)	< 0.001	(1.260–1.265)
*Social group*			
Others (Ref.)			
Other Backward Castes	0.77 (0.001)	< 0.001	(0.768–0.769)
Scheduled Tribes	0.67 (0.001)	< 0.001	(0.673–0.676)
Scheduled Castes	0.81 (0.001)	< 0.001	(0.805–0.807)
*Level of education*			
No education (Ref.)			
Primary	1.15 (0.001)	< 0.001	(1.146–1.150)
Secondary and above	0.91 (0.001)	< 0.001	(0.911–0.913)
*Wealth Index*			
Richest (Ref.)			
Rich	0.85 (0.001)	< 0.001	(0.853–0.855)
Middle	0.73 (0.001)	< 0.001	(0.726–0.731)
Poor	0.63 (0.000)	< 0.001	(0.625–0.627)
Poorest	0.58 (0.000)	< 0.001	(0.581–0.582)
*Working*			
No (Ref.)			
Yes	1.05 (0.001)	< 0.001	(1.053–1.056)
*Marital status*			
No (Ref.)			
Yes	2.22 (0.001)	< 0.001	(1.216–1.221)
*Children ever born*			
No Child (Ref.)			
One Child	1.84 (0.001)	< 0.001	(1.833–1.838)
Two or more Children	2.18 (0.001)	< 0.001	(2.179–2.184)
*Menopause*			
No (Ref.)			
Yes	1.74 (0.002)	< 0.001	(1.738–1.746)
*Tobacco use*			
No (Ref.)			
Yes	1.15 (0.001)	< 0.001	(1.145–1.150)
*Alcohol consumption*			
No (Ref.)			
Yes	1.02 (0.003)	< 0.001	(1.018–1.029)
*Waist–Hip ratio*	11.21 (0.032)	< 0.001	(11.147–11.271)
*Diet*			
Healthy (Ref.)			
Unhealthy	1.11 (0.001)	< 0.001	(1.110–1.112)

From the results, for an increase in age by a year, the risk of multimorbidity was 1.05 (95% CI 1.048–1.048) times. Similarly, the odds of having multimorbidity was 0.73 (95% CI 0.725–0.727) times among the rural participants than their urban counterparts. For women with primary education completed, the odds of having multimorbidity was 1.15 (95% CI 1.146–1.150) times than for women with no education. However, those with secondary and higher education odds was 0.91 (95% CI 0.911–0.913) times risk than those with no education. Further, for women from the poorest class, the odds of having multimorbidity was 0.58 (95% CI 0.581–0.582) times than for women from an affluent class. The odds of having multimorbidity for working women was 1.05 (95% CI 1.053–1.056) times than for unemployed women. In case of married women, the odds of having multimorbidity was 2.22 (95% CI 2.216–2.221) times than their unmarried counterparts. Furthermore, women with ‘two or more children’ and 'only one child’, the odds of having multimorbidity was 2.18 (95% CI 2.179–2.184) and 1.84 (95% CI 1.833–1.838) times when compared to those with no children. Women who have reached menopause had an odd of 1.74 (95% CI 1.738–1.746) times than those who had not reached menopause. Similarly, women who were using tobacco had an odds of having multimorbidity 1.15 (95% CI 1.145–1.150) times than those who did not use any tobacco.

When it comes to alcohol consumption, those women who have consumed alcohol had odds of having multimorbidity 1.02 (95% CI 1.018–1.029) times than those who did not consume alcohol. Lastly, for a one-unit increase in the waist-hip ratio, the odds of having multimorbidity was 11.21 (95% CI 11.147–11.271) times. And, women following an unhealthy diet had odd of having multimorbidity 1.11 (95% CI 1.111–1.113) times than those who did follow a healthy diet.

Table 4 shows the adjusted ordered logistic regression analysis of chronic disease scores among reproductive-aged women regressed upon various individual and household characteristics from National Family Health Survey-5, India, 2019–2021.

**Table 4:** Ordered logistic regression analysis of chronic disease score among reproductive-aged women, National Family Health Survey (NFHS-5), India, 2019–2021

Correlates	Adjusted relative risk ratio (ARRR)	*p*-value	95% Confidence interval
*Age (in years)*	1.04 (0.000)	< 0.001	(1.042–1.043)
*Place of residence*			
Urban (Ref.)			
Rural	0.90 (0.001)	< 0.001	(0.900–0.903)
*Religion*			
Hindu (Ref.)			
Muslim	1.05 (0.001)	< 0.001	(1.049–1.052)
Others	1.10 (0.001)	< 0.001	(1.094–1.099)
*Social group*			
Others (Ref.)			
Other Backward Castes	0.86 (0.001)	< 0.001	(0.856–0.858)
Scheduled Tribes	0.86 (0.001)	< 0.001	(0.860–0.863)
Scheduled Castes	0.94 (0.001)	< 0.001	(0.942–0.944)
*Level of education*			
No education (Ref.)			
Primary	1.28 (0.001)	< 0.001	(1.276–1.280)
Secondary and above	1.31 (0.001)	< 0.001	(1.307–1.311)
*Wealth Index*			
Richest (Ref.)			
Rich	0.94 (0.001)	< 0.001	(0.939–0.942)
Middle	0.86 (0.001)	< 0.001	(0.856–0.859)
Poor	0.78 (0.001)	< 0.001	(0.779–0.782)
Poorest	0.76 (0.001)	< 0.001	(0.754–0.757)
*Marital status*			
No (Ref.)			
Yes	1.23 (0.001)	< 0.001	(1.226–1.232)
*Working*			
No (Ref.)			
Yes	1.01 (0.001)	< 0.001	(1.008–1.011)
*Children ever born*			
No Child (Ref.)			
One Child	1.06 (0.001)	< 0.001	(1.053–1.058)
Two or more Children	1.07 (0.001)	< 0.001	(1.070–1.075)
*Menopause*			
No (Ref.)			
Yes	0.93 (0.001)	< 0.001	(0.930–0.935)
*Tobacco consumption*			
No (Ref.)			
Yes	0.99 (0.001)	0.001	(0.994–0.998)
*Alcohol consumption*			
No (Ref.)			
Yes	0.93 (0.002)	< 0.001	(0.930–0.935)
*Waist–hip ratio*	10.22 (0.029)	< 0.001	(10.163–10.277)
*Diet*			
Healthy (Ref.)			
Unhealthy	1.10 (0.001)	< 0.001	(1.094–1.097)

From the results, for an increase in age by a year, the risk of multimorbidity was found to be increasing by 4% (95% CI 1.042–1.043), given the other variables are held constant. Similarly, for rural residents, the risk of multimorbidity lesser by 10% (ARRR = 0.90; 95% CI 0.900–0.903) than urban residents, given the other variables are held constant. Further, for women with primary education completed, the risk of multimorbidity was found to be increasing by 28% (95% CI 1.276–1.280) than for women with no education. Whereas, those with secondary and higher education the risk was found to be increasing by 31% (95% CI 1.307–1.311) than those with no education, given the other variables are held constant. Further, for women belonging to the poorest class, the risk of multimorbidity was 24% (95% CI 0.754–0.757) lower than women who belong to an affluent class, given the other variables are held constant. For working women, the risk of multimorbidity was found to be increasing by 1% (95% CI 1.008–1.011) than unemployed women, given that the other variables are held constant. However, for married women, the risk of multimorbidity was found to be increasing by 23% (95% CI 1.226–1.232) than their counterparts, given that the other variables are held constant. For women who had two or more children and only one child, the risk of multimorbidity was found to be increasing by 6% (95% CI 1.053–1.058) and 7% (95% CI 1.070–1.075) compared to those with no children, given the other variables were held constant. Furthermore, women who have reached menopause, the risk of multimorbidity was found to be decreasing by 7% (ARRR = 0.93; 95% CI 0.930–0.935) than those who did not reach menopause, given the other variables are held constant.

Similarly, those women who have consumed alcohol the risk of multimorbidity was found to be decreasing by 7% (95% CI 0.930–0.935) than those who do not drink alcohol, given the other variables are held constant. Whereas, for a one-unit increase in the waist-hip ratio, the risk of multimorbidity was found to be increasing by 92.2% (95% CI 10.163–10,277), given the other variables were held constant. Finally, women following an unhealthy diet, the risk of multimorbidity was found to be increasing by 10% (95% CI 1.094–1.097) than those who follow a healthy diet, given the other variables were held constant.

## Discussion

The study suggests that roughly half of reproductive-aged women suffer from single morbidity, and one-fourth are affected with multimorbidity. Which somewhere aligns with the findings of the previous studies [1]. However, some studies have reported less prevalence probably because those were based upon half a decade old datasets, i.e., NFHS's fourth-round held in 2015–2016 [17, 33]. The average age of women with single morbidity is 30 years old, while the average age is just 35 years when single morbidity shifts to multimorbidity. Women with single morbidity can develop multimorbidity in as little as 6 years.

In India, rural women have less proportion who are suffering from multimorbidity than urban women, which can be ascertained by the fact that rural women have less accessibility to better health services and less cognizance of self-health condition [34]. Most of the chronic conditions in the study are self-reported, which perhaps underestimates the prevalence of chronic condition/s. In a very recent study, this phenomenon has been established that women in rural areas are less aware of NCDs and related repercussions [35]. The present study further suggests that the level of education has a strong inverse relationship with the number of chronic diseases a woman [1, 9, 13, 17, 33–37]. Furthermore, multimorbidity among those belonging to the affluent families has the highest proportion, perhaps due to obesity, dietary pattern, and physical inactivity [38]. Married women have a higher proportion of multimorbidity. Consistent with various other researches which support the current study findings [39, 40]. Parity and menopause are strongly associated with NCDs-related multimorbidity, with similar results established in previous studies [1, 9, 17]. Menopausal women tend to have low estrogen levels, impairing social and biological well-being [41]. This reduction in estrogen level promotes the risk of multimorbidity among reproductive-aged women [1]. These findings are in line with other studies as well [15, 23].

Similarly, the mean WHR for women, irrespective of the number of chronic conditions related to multimorbidity, is greater than the cut-off value of 0.85. Similar findings can be seen in other studies as well [1].

Northern India has the highest proportion of reproductive-age women suffering from chronic multimorbidity, followed by Central India. However, the proportion of women suffering from single morbidity is the highest in Southern and Central India. A study in Madhya Pradesh, a part of the central region of India, asserts that there is a shallow dietary diversity and high food insecurity, which can be attributed to the high level of single and multimorbidity among women of the reproductive age in the region [42]. This is perhaps a consequence of the absence of dietary diversity and food security in India which varies substantially across the nation [42–46]. Hence, after decrypting the factors that play a pivotal role in increasing the prevalence of multimorbidity among women are the region, working status, age of an individual, BMI, the level of education.

Further analysis highlights age as a significant predictor. With age, women go through various natural physiological transformations that promote multimorbidity in some way [41]. Apart from age, significant risk factors that came out loud are individual factors, including WHR, menopause, tobacco, alcohol consumption, and dietary habits, which significantly affect the multimorbidity status among women in the reproductive age group. These findings are again aligned with prevailing literatures [9, 35, 47]. WHR is significantly associated with central obesity, and it eventually results in chronic diseases [41, 48]. Hence, awareness programs are required to combat and reduce many avoidable cases due to modifiable risks. Dietary choices were found to be strongly linked to the condition/s under study among women.

Anemia and obesity are two common chronic illnesses among the study sample, which are a direct consequence of poor dietary habit [11, 49–51]. Hence, ensuring better dietary habits and dietary diversity among reproductive-aged women is essential. Further, social factors such as the women's education which is inversely proportional to the chronic condition related to multimorbidity, working status, parity, wealth index, and religion, are significantly associated with the women's morbidity status. Various previous studies have produced likewise results [17, 33, 52]. These findings add up to our prevailing knowledge in ascertaining the health status of reproductive-aged women in the nation.

The present study's strengths lie in the fact that it is based on the most recent round of the nationally representative survey i.e., NFHS series held in 2019–2021 with a robust sample size. Moreover, it has established some significant inversely or directly associated risk factors with the number of chronic conditions related to morbidity among women in the reproductive age group. Besides the strengths mentioned, there are a few limitations. Firstly, limited information on the number of chronic diseases in the sample is available. Secondly, using a cross-sectional data comes with its own set of constraints, such as to establish causality. Third, factors such as individual stress level and family/parental history of any chronic disease were not considered due to the unavailability of the information. Lastly, prevalence has been computed using the data collected by asking women if she is suffering from any of the eight chronic diseases, which might give estimates different than the actual scenario.

## Conclusions

The present study hints that women in the reproductive age group are at very high risk of multimorbidity. The mean age of reproductive-aged women with single morbidity to suffering from two or more chronic diseases is 30 to 35 years. Hence, policymakers and service providers should focus on those aged 30 to 35, as these are crucial ages where appropriate intervention would perhaps forestall this shift. Furthermore, the primary focus is required among those living in a low socio-economic settlement. Again, it is imperative to make them aware of how and what behavioural aspects induce multimorbidity and its implications and long-term consequences. So that self-reporting and measured prevalence converge hence minoring reporting errors.

All the existing programs and policies focus on the elderly population in terms of awareness and facilitating them with better health services. However, right now, one should also prioritize the emerging multimorbidity burden among reproductive-aged women. Further, emerging issues of obesity among women from economically better off households where diet-based NCDs are prevailing should be prioritized. If this target group continues to be overlooked, India might experience a multimorbidity epidemic in the coming years. Increasing multimorbidity burden among reproductive-aged women has implications at various levels of the life course of mother and child. Thus, it is essential for policymakers to take appropriate actions to control the mounting danger of multimorbidity.

## Data Availability

The study utilizes a secondary source of data which is available on request and is available in the public domain through: https://dhsprogram.com/data/dataset/India_Standard-DHS_2020.cfm?flag=1.

## Abbreviations

CDS: Chronic Disease Score
LMICs: Low-and-Middle-Income Countries
NCDs: Noncommunicable Diseases
WHO: World Health Organization
